# Inferring Tripartite Associations of Vector-Borne Plant Pathogens Using a Next-Generation Sequencing Approach

**DOI:** 10.3390/pathogens14010074

**Published:** 2025-01-14

**Authors:** Ava M. Gabrys, Christopher H. Dietrich, Valeria Trivellone

**Affiliations:** 1 Department of Biology, The Pennsylvania State University, University Park, PA 16802, USA; avagabrys@gmail.com; 2 Illinois Natural History Survey, Prairie Research Institute, University of Illinois at Urbana-Champaign, Champaign, IL 61820, USA; chdietri@illinois.edu

**Keywords:** phytoplasma, vector-borne pathogens, cophylogeny, leafhopper, diet breadth

## Abstract

Phytoplasmas are a group of plant-pathogenic, cell-wall-less bacteria vectored primarily by leafhoppers (Hemiptera Cicadellidae), one of the most diverse families of insects. Despite the importance of documenting associations between phytoplasmas, their insect vectors, and plant hosts to prevent disease outbreaks, such knowledge is currently highly incomplete and largely neglects the diversity of the system in natural areas. Here, we used anchored hybrid enrichment (AHE) to recover the DNA of five plant genes (*rbcL*, *matK*, *ITS1*, *ITS2*, and *trnH-psbA*) in 58 phloem-feeding leafhoppers from around the world that had previously tested positive for phytoplasma infection. Using BLASTn and a strict filtering approach, we assigned taxonomic classifications to the plant sequences and tested for cophylogenetic signals between potential Deltocephalinae leafhopper vectors and their associated plants. We observed incongruence between plant and insect phylogenies. Many leafhopper species, including presumed grass specialists, fed on distantly related plant lineages; 66% of sampled leafhoppers fed on plants from at least two different orders. By disentangling phytoplasma–leafhopper–plant interactions, we identify locations at risk of phytoplasma disease outbreaks. Furthermore, the observed wide diet breadth raises questions about how phytoplasma infection may manipulate the feeding preference of their insect host and helps fill the gaps in understanding the ecology and diversification of the tripartite association.

## 1. Introduction

Phytoplasmas are a group of plant-pathogenic, cell wall-less bacteria that infect hundreds of known plants around the world. They are vectored in a persistent, propagative manner by phloem-feeding insects, principally leafhoppers (Hemiptera Cicadellidae). With over 23,000 described species, leafhoppers are among the most diverse groups of insects, and the majority of the phytoplasma vectors are in the (largest) subfamily, Deltocephalinae [1]. Unfortunately, knowledge of associations between phytoplasmas, their insect vectors, and plant hosts remains highly incomplete. Documenting phytoplasma–vector–plant associations is critical to understanding the evolutionary history of the system and thus predicting and preventing emerging outbreaks [2]. However, the documented associations almost exclusively focus on agroecosystems, with screening for phytoplasmas largely limited to previously known insect vectors or insects strictly collected on the target crops. This narrow focus has left significant gaps in our understanding, as it has been noted that vectors remain unidentified for at least half of the previously documented phytoplasma groups. Furthermore, the host plants associated with potential Auchenorrhyncha vectors are poorly documented, underscoring the need for broader ecological and biological studies [3].

Leafhoppers complete their lifecycle and often lay eggs and overwinter on particular vascular plants but may be associated with various plants as feeding hosts. Leafhoppers associated with non-persistent, spatially and temporally variable plants are more likely to have a wider diet breadth, have multiple generations per year, and exhibit greater dispersal abilities [4,5]. Leafhoppers and their host plants have been evolving together for over 260–280 million years [6], and leafhopper diet breadth has changed over time. At a broader taxonomic resolution, the diversification of the leafhopper subfamily Deltocephalinae, in which grass/sedge specialization is an evolutionary conserved trait, is thought to have coincided with the spread of grasslands around the world [7]. Besides phylogenetic conservatism, other processes driving the evolution of insect–plant associations may explain the expansion and contraction of the host range at ecological and evolutionary time scales. For example, vector-borne plant pathogens may manipulate host biological traits (e.g., behavior, physiology, and morphology [8,9,10,11]) to enhance their transmission and dispersal [12], yielding the rapid expansion of diet breadth to include multiple plant species [13]. To illustrate, *Macrosteles quadrilineatus*, a notorious vector of aster yellows disease, transmits multiple phytoplasma species and its diet breadth is thought to have expanded under the influence of several factors [14]. Likewise, *Euscelis incisus* is known to host several strains of phytoplasmas under experimental conditions, and its association with a broad range of habitat types facilitates its role as a carrier of multiple phytoplasmas in the field, maintaining latent infections across the landscape [15].

Traditional approaches to reconstructing insect diet breadth, such as field observations and mesocosm experiments, are prone to inaccuracies and are time-consuming and labor-intensive. Indirect observations of associations with particular plant species, inferred from sweeping the vegetation, can be misleading because leafhoppers are highly mobile and thus may be collected on plants that are not their food or oviposition hosts. In captivity, leafhoppers may often feed and develop on host plants upon which they are never found in nature. Analyzing the gut content of specimens collected from natural and anthropogenic environments to characterize ingested plant material is a robust technique for overcoming challenges in understanding feeding behaviors and ecological interactions. This method provides direct evidence of associations with plants, avoids biases inherent in observational studies, and provides insights into plant–vector or plant–phytoplasma relationships critical for epidemiological research. DNA from digested plant material in the gut has been extensively studied in leaf-chewing insects (e.g., [16,17,18]). Because they feed primarily on plant vascular fluids, sap-sucking insects might be expected to acquire less plant DNA during feeding than insects that chew on and ingest leaf or stem tissues. However, recent studies have demonstrated that piercing-sucking insects, such as leafhoppers, often retain plant DNA, presumably ingested during feeding, that can be detected and characterized using Sanger and next-generation sequencing approaches [19,20,21]. Because phloem-feeding insects may also ingest phytoplasmas, which reside in the phloem of infected plants, screening DNA extracted from phloem-feeding insects can reveal previously undocumented associations between these insects, their food plants, and the phytoplasmas infecting those plants. This may help fill the major remaining gaps in our knowledge of these tripartite interactions. 

In this study, we used a hybrid enrichment-based approach to infer relationships between leafhoppers previously obtained in worldwide biodiversity surveys, phytoplasmas, and their host plants. We sequenced and assessed the quality of five standard plant barcode genes—ribulose 1-5 bisphosphate carboxylase subunit (*rbcL*), maturase k (*matK*), internal transcribed spacers 1 and 2 (*ITS1* and *ITS2*), and intergenic spacer *trnH-psbA* [22]—in 75 DNA samples extracted from individual leafhoppers that previously tested positive for phytoplasma using qPCR. Using strict filtering criteria, we reconstructed the diets of 58 insects and tested for coevolution between these plants and their associated vectors.

## 2. Materials and Methods

### 2.1. Leafhopper Samples, DNA Extraction, qPCR, and Anchored Hybrid Enrichment Sequencing

From 2018 to 2022, we conducted a preliminary screening of 634 leafhopper specimens selected from a comprehensive collection at the Illinois Natural History Survey. These specimens, collected from natural habitats during more than 20 years of biodiversity sampling expeditions, represent all major biogeographic regions. DNA was extracted from individual leafhoppers using the Qiagen DNeasy Blood and Tissue Kit (Qiagen, Germantown, MD, USA), following the protocol outlined by Trivellone et al. (2022) [23]. To detect the presence of phytoplasmas in individual insects, qPCR targeting the 16S ribosomal gene was performed, as described by Angelini et al. (2007) [24]. Phytoplasma-positive samples were then analyzed using a newly developed anchored hybrid enrichment (AHE) probe kit, which includes 58,000 DNA probes targeting 129 phytoplasma genes conserved across 50 partial or complete phytoplasma genomes, and 5 plant genes. This approach enabled the acquisition of additional DNA sequence data. The quality of the probe design was validated using existing phytoplasma genomic resources, following the procedures described by Lemmon et al. (2012) [25].

### 2.2. Phytoplasma and Plant DNA Detection and Characterization

The data from phytoplasma genes have been analyzed in separate studies [23,26] and here we report the results of phytoplasma identification using the 16S rRNA gene (when available) and 6 additional loci (tuf, secA, secY, rpIV, rpsC, and groEl). The AHE probe kit gathered data from plants for the following genes: *rbcL*, *matK*, *ITS1*, *ITS2*, and *trnH-psbA*.

As identification inaccuracies have proved more common with short sequences [20,27], we employed strict filtering to identify plant DNA at the genus level or higher. Each sequence was run through BLAST v.2.13.0 [28] against the Virdiplantae kingdom in the NCBI nucleotide database. Results for each sequence were initially filtered to require a percent identity greater than 98% and query coverage of 97%, followed by filtering for within 1% percent identity of the top hit. Taxonomic classification (genus, family, or order) was given at the lowest level in which >95% of the filtered results were in agreement. To limit inaccuracies caused by short sequences, taxonomic assignments for incomplete sequences <220 bp long were only considered if they could be corroborated with at least one other sequence from that sample (see Data for R scripts). Taxa names were obtained using the *efetch* function in the “reutils” R package. Different DNA sequences from the same insect were processed as individual samples, with summarized results combined at the end. To explore how likely the recovered sequences encompass the true diversity of ingested plants, we assessed the relationship between the number of recovered plant sequences and leafhopper diet breadth using a linear regression model.

### 2.3. Insect–Plant Cophylogenetic Analysis

The insect phylogeny for samples with genus-level plant results was obtained from Cao et al., 2022 [7] by pruning the tree using the “castor” R package [29]; a total of 35 taxa were included. This tree included the molecular data from 13 insect specimens screened in our study. For the remaining specimens, tips were selected for pruning by matching to the same species (9 specimens) or genus identification (13 specimens) (Supplementary Table S3). Nine specimens with ingested plant data identified at the genus level were excluded due to the absence of a near equivalent from the published phylogeny or being the same species as another sample that encompassed its associations. To obtain the plant phylogeny, genus-level results for long or corroborated sequences were further classified to the species level. A species was classified for a genus when multiple sequences yielded the same species identification (most results within 1% of the highest percent identity) or, in the case of no agreement, to the species with the highest percent of filtered results (Supplementary Table S3). In this way, species with an uncertain species-level assignment would be retained within the same genus. The phylogeny was built using the GBOTB.extended.TPL mega tree of vascular plants [30,31]—which contained 48/63 species—in V.PhyloMaker2 [32,33] with Scenario 3. Any multifurcating nodes were made bifurcating using the “ape” package [34]. Associations to the non-vascular plants *Thamnobryum* (mosses) for two samples and *Klebsormidium* (algae) for one sample were excluded. For cophylogenetic analyses, a random tanglegram partitions (Random TaPas) approach [35] based on the global fit method was used to evaluate the cophylogenetic signal between the insect (35 species) and plant (63 species) phylogenies. Both algorithms (maximum congruence and maximum incongruence) were applied with multiple association correction (res.fq = TRUE) and subtraction of frequencies in extreme percentiles (diff.fq = TRUE), respectively. We chose PACo as the global fit method with symmetric = TRUE, as we assumed that leafhopper diversification is not driven by that of the plant host. These algorithms reveal the taxa and clades that contribute most strongly to overall congruence or incongruence. The cophylogenetic tanglegram was built using the R package RTapas [35,36].

## 3. Results

### 3.1. Inferring the Host Plants of Potential Leafhopper Vectors Using AHE Data

From a total of 634 specimens, 96 samples that tested positive for the presence of phytoplasmas were submitted for sequencing. For the five targeted plant loci—*rbcl*, *matK*, *ITS1*, *ITS2*, and *trnH-psbA*—a combined total of 939 sequences were recovered (Table 1) from 75 samples (corresponding to 68 specimens; DNA from five specimens was submitted for sequencing twice, and DNA from one specimen was submitted three times). In total, 56 species (58 individual leafhoppers, Supplementary Table S1) yielded filtered results, and these were used for further analysis. Using data obtained for six different phytoplasma loci (16Sr or other informative housekeeping genes), a total of 32 phytoplasma strains were detected in the bodies of the leafhoppers screened in this study.

#### 3.1.1. Variation in Sequence Recovery Among Loci

*rbcl* was the best-represented locus, recovered in 84% of samples and comprising 35% of recovered sequences, while *trnH-psbA* was captured the least, making up only 3% of recovered sequences. With an average (avg.) sequence length of only 144 bp, *ITS1* had the highest rate of complete sequence recovery (10.7%) and was recovered in the second-highest proportion of samples (70.7%). Likewise, *ITS2* had shorter (avg. 169 bp) sequences and was similarly recovered in 66.7% of samples. On the other hand, *matK* and *rbcL* had comparatively long sequences (255 and 246 bp, respectively), and no sequences were recovered in full (Table 1). 

Following classification with BLASTn, *ITS2* had the highest percent of sequences confidently identified to the genus level (wherein 95% of results within 1% of the top percent identity are in agreement, see Methods), consistent with previous results indicating that this locus has sufficient discriminatory power at lower classification levels [37]. However, conflicting results for this locus were more likely to be in different families and orders, as suggested by the lowest family-level and order-level support across all loci (65.1% and 86.7%, respectively). In contrast, less than half of the *matK* results were well-supported at the genus level, but any divergence of classification was within the same family and order. Similarly, for *rbcL*, a high proportion of sequences have low genus-level support but a high proportion of BLAST results were in agreement at the family (88%) and order (95.7%) levels, consistent with *rbcL* being a slowly evolving gene [38].

#### 3.1.2. Influence of Sequence Count on Identified Diet Breadth

For cases where multiple plant sequences were obtained from a single insect, these either corroborated the identification of a single food plant species or suggested that the insect fed on more than one plant species (Supplementary Figure S1). For example, the vast majority (125/129) of the sequences attributed to the sample with the highest number of recovered sequences (P001_WC11, *Coganus breviatus* collected in South Africa) were short (<220 bp sequences) and filtered to only three resulting plant species. In contrast, the sample with the second highest number of sequences (P001_WF04, *Osbornellus auronitens* collected in Switzerland), with 98 recovered plant sequences, also recovered the longest sequences (47) and well-supported identifications of different potential food plant species (18). 

The linear regression of the number of well-supported plant identifications versus total recovered sequences in the sample suggests that the overall sequence count minimally drives the diet breadth inferred in our results (R^2^ = 0.36, slope = 0.09). In contrast, the number of recovered long sequences has a stronger impact on the number of plant identifications supported for each specimen (R^2^ = 0.82, slope 0.42) using our filtering criteria.

### 3.2. Tripartite Vector–Plant–Phytoplasma Associations

**Figure 1:**
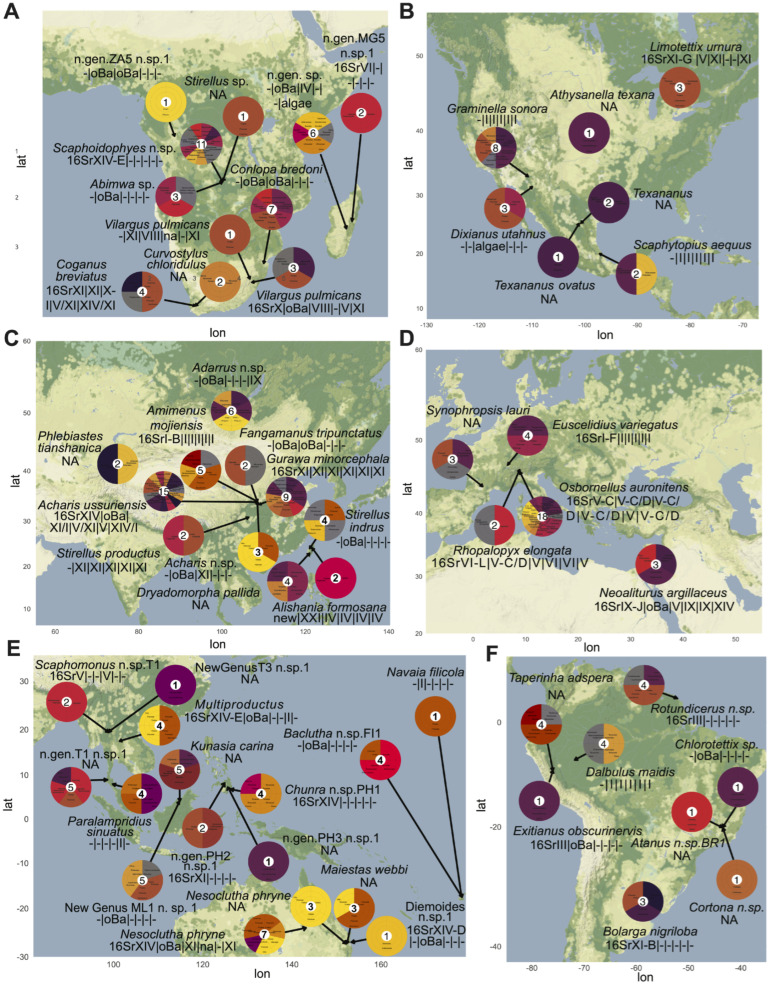
Vector–plant–phytoplasma associations for 58 leafhoppers mapped across six geographic areas: Africa (**A**), North America (**B**), North and East Asia (**C**), Europe and the Middle East (**D**), Southeast Asia and Oceania (**E**), and South America (**F**). For each leafhopper, unique results to a plant order, family, or genus are each represented by an equal proportion of the pie, and the center number represents the total number of these unique results. A leafhopper may have multiple hits matching a particular higher taxon (order/family) and a lower taxon (family/genus) within that higher taxon, indicating that multiple food plants are possible within that higher taxon but cannot currently be distinguished given the available data. Phytoplasma classification across loci is listed in the format 16Sr|Tuf|SecA|SecY|rpIV.rpsC|groEl, where “oBa” indicates other bacteria, “-” indicates classification unclear or not available on the corresponding locus, and “NA” indicates no classification data available.

Of the 58 leafhoppers with recovered plant sequences from the 96 that tested positive for the presence of phytoplasma, 38 had results classified to more than one order and 25 had results classified to at least three orders, indicating wide diet breadths for nearly half of the potential phytoplasma vectors. For 45 specimens, at least one result classified to the genus level was retrieved. The summarized and filtered results for each specimen can be found in Supplementary Table S1 and the summarized BLAST results for each sequence can be found in Supplementary Table S2. The inferred tripartite associations are summarized in Table 2 and mapped across six geographic areas in Figure 1, including the number of unique plant classifications in the pie graph and the phytoplasma strain identified in the same specimen using six different loci (housekeeping genes).

#### 3.2.1. Africa

Plant sequences were recovered from 11 specimens collected across sub-Saharan Africa. Nine specimens were from five tribes in Deltocephalinae (Stenometopiini, Scaphoideini, Bonaspeiini, Paralimnini, and Selenocephalini) (Figure 1A, Table 2). A total of at least six phytoplasma strains were detected. *Scaphoidophyes* sp. (Scaphoideini) in Northwestern Province, Zambia carried 16SrXIV-E group phytoplasma with ten well-supported hits encompassing nine different plant orders: Fabales (two results in Fabaceae, including *Brachystegia*), Asterales, Gentianales, Laurales, Sapindales, Liliales, Zingiberales, Lamiales, and Solanales. Collected at the same site, *Abimwa* sp. also fed on *Brachystegia* (tropical mimbo trees) and, interestingly, two *rbcl* sequences support feeding on *Klebsormidium* (filamentous algae) (Supplementary Table S2). *Stirellus* sp., thought to be a grass specialist, indeed fed on Poales (*Cenchrus*). However, such a result was only supported by one sequence with >98% identity, and the recovery of only two sequences limits the identification of a potentially wider diet breadth (Supplementary Table S2, Supplementary Figure S1).

Sequences were recovered from four South African specimens. Two *Vilargus pumilicans* specimens, a previously unknown potential vector carrying phytoplasma with ambiguous classification (16Sr-XI, -X, or -VIII groups) from the KwaZulu-Natal province, corroborated feeding on grasses in Poaceae; additional recovered sequences in one specimen suggested that these reportedly grass-specialist leafhoppers [39] feed on Asterales (Asteraceae) and Malvales (Malvaceae) as well. Among two leafhoppers of the tribe Bonaspeiini, only plants in the family Moraceae, including *Morus* (mulberries), were identified in *Curvostylus chloridulus*, while *Coganus bredoni*, collected in the Western Cape Province, fed on Poales, Zingiberales, and Apiales (Figure 1A, Table 2).

Two new leafhopper genera collected in this expedition belong to the tribe Scaphoideini, which includes known vectors such as *Scaphoideus titanus* (vector of Flavescence dorée in Europe [40]) and *Obsornellus horvathi* (vector of aster yellows disease [41]). In a hunting reserve in Pool, the Republic of the Congo, one specimen of an undescribed genus (n.gen.ZA5) belonging to Scaphoideini was associated with the family Vitaceae (Vitales), although further plant identification was limited by the recovery of only two sequences (Supplementary Table S2, Supplementary Figure S1). In the Moramanga District, Madagascar, all four sequences recovered from a specimen from an undescribed genus, n.gen.MG5 belonging to the tribe Scaphoideini, were corroborated as Hypnales (mosses). Another specimen collected in Madagascar, belonging to the tribe Stenometopiini, had a very wide diet breadth, with associations to the orders of Cupressales, Rosales, Solanales, and Fabales, including the genera *Taxus* (Taxaceae), *Ulmus* (Ulmaceae), Capsicum (Solanaceae), and Glycine (*Fabaceae*) (Figure 1A, Table 2). 

Finally, diet was assessed for one species from the subfamily Ulopinae. Collected in the Mlawula Nature Reserve (Eswatini, southeast Africa), *Conlopa bredoni* had six supported results matching four different orders (Commelinales, Fabales, Poales, and Lamiales). This species was found in association with non-phytoplasma bacteria (Figure 1A, Table 2).

#### 3.2.2. North America

Our results were recorded for seven leafhoppers representing six tribes (Phlepsiini, Deltocephalini, Chiasmini, Opsiini, Scaphytopiini, and Limotettigini) in Deltocephalinae collected in North America (Figure 1B, Table 2). Four of them belong to genera known to include vectors of 16SrI-related phytoplasma strains (*Texananus*, *Graminella*, *Limotettix*, and *Scaphytopius*). A total of at least three phytoplasma strains were detected. Two infected insects in the genus *Texananus* (Phlepsiini), sampled from Coahuila (Mexico), corroborated results only to Asteraceae, with one sample specifically suggesting feeding on native *Ambrosia*. A specimen in the tribe Deltocephalini, *Graminella sonora*, had well-supported results for eight different taxa (orders Asterales, Caryophyllales, Piperales, Poales, and Rosales) while carrying a 16SrI-related phytoplasma strain. *Scaphytopius aequus* (Scaphytopiini) was collected in a Mexican rainforest (Veracruz) and our results indicated associations with plants in the Solanaceae and Caryophyllaceae families. This specimen also tested positive for a 16SrI-related phytoplasma strain. The sedge feeder *Limotettix urnura* (Limotettigini), collected in Ontario (Canada), fed on multiple taxa in Poales and was infected with a phytoplasma belonging to the 16SrXI-G subgroup. *Dixianus utahnus* (Opsiini), a previously unknown vector found carrying unclassified phytoplasma, fed on Poales (Poaceae and *Saccharum*) and Fabales (Fabaceae) (Figure 1B, Table 2). Lastly, a single sequence recovered from *Athysanella texana* (Chiasmini) suggests it at least feeds on *Silene* (Supplementary Table S2).

#### 3.2.3. North and East Asia

Plant sequences were recovered from 11 specimens in Deltocephalinae across seven tribes (Paralimnini, Stenometopiini, Chiasmini, Drabescini, Hecalini, Scaphoideini, and Opsiini) (Figure 1C, Table 2). A total of at least eight phytoplasma strains were detected in specimens from this continent. In mainland China, plant sequences were recovered from five species in grass-specialist lineages; although each fed on Poaceae, the data also suggest feeding on plants of other orders. Four of them were collected on a grassland in a Forest Natural Reserve (Shaanxi). *Acharis ussuriensis* (Paralimnini) yielded DNA from the second-highest number of different plant taxa in this study (15), belonging to nine different orders: Roasales, Asparagales, Pinales, Sapindales, Apiales, Fagales, Asterales, Laureales, and Poales. Another yet undescribed species in the genus *Acharis* was found feeding on Poaceae (Poales) and Vitales (Vitaceae and *Vitis*), and *Fangamanus tripunctatus* (Hecalini) was identified as feeding on Poaceae (Poales) and Malvaceae (Malvales). One specimen in the tribe Chiasmini, *Gurawa minorcephala*, was identified as carrying a 16SrXI-related phytoplasma strain and was associated with plants belonging to eight different orders: Asterales, Caryophyllales, Hypnales, Poales, Pottiales, Rosales, and Ophioglossales, including the genera *Youngia*, *Rubus*, *Stellaria*, and *Botrychium*. In Shaanxi as well, *Amimenus mojiensis* (Scaphoideini) fed on five plant orders while carrying 16SrI-B phytoplasma: Dipsacales, Poales (including *Saccharum*), Sapindales (*Ailanthus*), and Myrtales (*Miconia*). *Stirellus productus* (Stenometopiini) collected in Sichuan (mainland China), also carrying a 16SrXI-related phytoplasma strain, was identified as feeding on Poales (Poaceae) and Fagales (Fagaceae) (Figure 1C, Table 2).

In Taiwan, *Alishania formosana* (Opsiini) carrying phytoplasma classified as a new group [23] was associated with sequences of plants identified as Fagales, including Fagaceae. Solanales was identified in *Stirellus indrus* (Stenometopiini), specifically the genus *Solanum*, along with *Avena* (Poaceae) and Pteridaceae (Polypodiales). The third species in Taiwan, *Dryadomorpha pallida* (Drabescini), had well-supported results to Ericales (including *Actinidia*), Rosales (*Cannabis*), and Caryophyllales (*Silene*) (Figure 1C, Table 2). 

The remaining two specimens belong to the tribe Paralimnini. *Phlebiastes tianshanica* was collected at Naryn Alabuga River (Kyrgyzstan) and was found to be associated with plant DNA from a species belonging to Apiaceae (Apiales) and a species in the genus *Solanum* (Solanaceae). An undescribed *Adarrus* leafhopper species collected in Mongolia was associated with the orders of Asterales, Caryophyllales, Vitales, and Sapindales (Figure 1C, Table 2). 

#### 3.2.4. Europe and the Middle East

We reconstructed diets for five vectors across five different tribes of Deltocephalinae (Scaphoideini, Cicadulini, Athysanini, Opsiini, and Fieberiellini) collected in Europe and the Middle East, all of which were associated with plants in multiple orders (Figure 1D, Table 2). A total of four phytoplasma strains were recorded. *Osbornellus auronitens* (Scaphoideini), an alien species for the Palearctic continent (native to North America) collected in south Switzerland (Stabio), had an abundant 98 recovered plant sequences (47 with length >220 bp) (Supplementary Table S2, Supplementary Figure S1) and the most well-supported classifications (18 plant taxa), with eight to the genus-level (*Silene*, *Cornus*, *Medicago*, *Trifolium*, *Corylus*, *Solanum*, and *Vitis*). This is the first time *O. auronitens* has been shown to carry 16SrV-C/D, commonly known as Flavescence dorée, phytoplasma in Europe. Nearby in the same country, *Rhopalopyx elongata* (Cicadulini) carried a phytoplasma belonging to the 16SrVI-L subgroup (corroborated on the 16Sr gene), and this is the first record of this genus being infected by a phytoplasma. This specimen was also associated with Hypnales (feather mosses) and Ginkgo, with classification to non-vascular plants supported by seven out of ten recovered sequences from three different loci (*rbcL*, *ITS1*, and *ITS2*) (Supplementary Table S2). Lastly, in Switzerland, *Euscelidius variegatus* (Athysanini), collected on the ground cover vegetation surrounding a vineyard plot in Canton Vaud, feeds on the orders Fabales (including *Medicago*) and Caryophyllales (including *Silene*) while carrying a phytoplasma strain belonging to 16SrI-F subgroup. This leafhopper species is a known competent experimental vector of Flavescence dorée [42] and other phytoplasma groups (e.g., [43,44]). The other two specimens each had DNA matching three different plant orders. In particular, *Synophropsis lauri* (Fieberiellini), infected with a variant of 16SrIII-U phytoplasma [23], was collected in Montans (France) and yielded plant DNA belonging to Ginkgoales (*Ginkgo*), Poales (Poaceae), and Caryophyllales (*Silene*). *Neoaliturus argillaceus* (Opsiini) belongs to a genus with several species known as competent vectors of phytoplasmas; in particular, *Neoaliturus haematoceps* was previously reported as a potential vector of a phytoplasma belonging to 16SrIX [45]. Here, we report for the first time *N. argillaceus*, collected from desert vegetation in Rishon LeZion (Israel), infected with 16SrI-J phytoplasma. Moreover, we detected DNA from the following plants: Asterales (*Cathamus*), Caryophyllales (Polygonaceae), and Lamiales (Verbenaceae).

#### 3.2.5. Southeast Asia and Oceania

Diets were reconstructed for 16 leafhoppers in Southeast Asia and Oceania, 15 of which belonged to six tribes across Deltocephalinae (Opsiini, Scaphoideini, Stegelytrini, Drabescini, Macrostelini, Deltocephalini, and Paralimnini). All but six were inferred to have fed on multiple orders (Figure 1E, Table 2). A total of eight phytoplasma strains were recorded. In Thailand, a leafhopper belonging to an undescribed genus (New Genus T3) in Opsiini only harbored DNA of a plant species in the family Caryophyllaceae, though the recovery of only two sequences limited the identification of any additional food plants (Supplementary Table S2, Supplementary Figure S1). With six recovered sequences, including four >220 bp, a new species of *Scaphomonus* (Scaphoideini) infected with a 16SrV-related phytoplasma strain was associated only with the order Hypnales (Supplementary Table S2). An undescribed genus of tribe Stegelytrini (n.gen.T1), collected near an agricultural area in the Nakhon Si Thammarat Province (Thailand), was found associated with Neckeraceae (Hypnales), Poaceae (Poales), and *Bidens* (Asteraceae). Nearby, *Paralampridius sinuatus* (Opsiini) fed on the families Poaceae (Poales), Pottiaceae (Pottiales), and Caryophyllaceae (Caryophyllales) while carrying a phytoplasma from the 16SrI group. The fairly large number of recovered sequences, with each yielding six sequences >220 bp, and the lack of overlap in plant identifications support distinct diets for these species. Lastly, a specimen in the genus *Multiproductus* (Paralimnini) was found infected with 16SrXIV-E-related phytoplasma, although this result was not confirmed by all the investigated genes (Table 1). This specimen was associated with a grass species in the genus *Saccharum* (Poaceae) and *Vitis* (Vitaceae) and well-supported with 41 recovered sequences (Supplementary Table S2). 

In the Philippines, one new genus in Scaphoideini (n.gen.PH2) was collected at a forested site in Bukidnon, feeding on native *Opocunonia* trees (Oxidales Cunoniaceae) and Poaceae while carrying a phytoplasma from the 16SrXI group. The other new genus collected in Batangas (Philippines) belongs to the tribe Drabescini and only had one recovered plant sequence, classified to the genus *Silene* (Supplementary Table S2). Lastly, we identified sequences in the orders Rosales (Moraceae, *Morus*, and *Prunus*) and Fabales (*Trifolium*) in a new *Chunra* species in the subfamily Eurymelinae that hosted a 16SrXIV-related phytoplasma strain (Figure 1E, Table 2). 

Two *Nesoclutha phryne* (Macrostelini), thought to be grass specialists [46], were collected in Queensland (Australia) with inconclusive phytoplasma classification of a strain belonging to the 16SrXIV/XI group. Eight recovered plant sequences resulted in classification to the Vitales order, while additional sequences from a second *N. phryne* specimen were identified as Poaceae (including *Tripogonella*), Ulmaceae, and Caryophyllaceae (*Silene*), as well as *Vitis* (Supplementary Table S2). Another Australian leafhopper specimen, *Maiestas webbi* (Deltocephalini), similarly fed on families Poaceae (*Saccharum*) and Vitaceae (*Vitis*). Lastly, a new species in *Diemoides* (Scaphoideini) fed on Solanales (Solanaceae) while carrying a phytoplasma from the 16SrXIV-D-related subgroup (Figure 1E, Table 2).

In Fiji, a new species in the genus *Balclutha* (Macrostelini) and *Navaia filicola* (Opsiini) both yielded plant sequences identified as Poaceae; *Balclutha* n.sp.FI1 also had sequences matching with Hypnales (including *Thamnobyrum*), while the recovery of only one sequence from *N. filicola* limits the identification of any further diversity in diet (Figure 1E, Supplementary Table S2, Supplementary Figure S1). This latter specimen of leafhopper was also found infected with a phytoplasma in the 16SrI group. Like the specimen of a new genus of Scaphoideini in Africa and *Rhopalopyx elongata* in Europe, the peculiar association with mosses is well-supported, where all but four of 57 recovered sequences spanning all five probed loci had best matches to plants of that order (Supplementary Table S2).

In Malaysia, a new genus in the tribe Opsiini (New Genus ML1), which includes competent vectors of many phytoplasma groups, was found associated with plant DNA from three orders: Sapindales (*Citrus*), Poales (*Saccharum*), and Malvales (Dipterocarpaceae). Additionally, *Kunasia carina*, remaining inconclusively defined as a potential vector, harbored plant material matching orders Caryophyllales (*Silene*), Myrtales (including *Syzygium*), and Pottiales (Pottiaceae) (Figure 1E, Table 2). The peculiar result for Pottiaceae (mosses) was supported by both *rbcL* (233 bp) and *ITS2* (168 bp) sequences (Supplementary Table S2).

#### 3.2.6. South America

We obtained results for eight leafhopper specimens belonging to different genera (*Atanus*, *Bolarga*, *Cortona*, *Chlorotettix*, *Dalbulus*, *Exitianus*, *Rotundicerus*, and *Taperinha*) representing six tribes in Deltocephalinae (Deltocephalini, Athysanini, Pendarini, Macrostelini, Chiasmini, and Bahitini) and one in Eurymelinae (Chiasmodolini), including four species not yet described (Figure 1F, Table 2). A total of four phytoplasma strains were recorded. Three of the new species were collected in a rural mountainous site in Minas Gerais (Brazil) and were found associated with single plant taxa: *Silene* for *Chlorotettix* sp. (Pendarini), *Salvia* for *Atanus* sp. (Athysanini), and the Pottiaceae family for *Cortona* sp. (Deltocephalini). Notably, only one sequence was recovered for *Chlorotettix* sp. and only two were recovered for *Cortona* sp., limiting the identification of potentially wider diet breadths (Supplementary Table S2). Like *Chlorotettix* sp., *Exitanus obscurinervis* (Peru) had results only for the plant genus *Silene* in the leafhopper’s first recorded association with phytoplasma group 16SrIII.

*Dalbulus maidis* (Macrostelini), collected in a forested area of Amazonas (Brazil), was found to be associated with *Citrus* (Rutaceae), *Musa* (Musaceae, bananas, and plantains), and a species in the genus *Nephrolepis * (Nephrolepidaceae). This specimen was also found to be infected with a 16SrI-related strain of phytoplasma. A new species in *Rotundicerus* (Chiasmodolini) collected in French Guiana was also found to be associated with plant species belonging to three orders: Poales (including *Saccharum*), Caryophyllales, and Cupressales, while being associated with a 16SrIII-related phytoplasma strain. In the tribe Bahitini, *Taperinha adspera* (Peru) fed on three orders: Dipsacales (*Sambucus*), Oxidales (including *Opocunonia*), and Myrtales (*Syzygium*). Lastly, in a grassland site near an agricultural area in Entre Ríos, Argentina, *Bolarga nigriloba* (Deltocephalini), carrying 16SrXI-B phytoplasma, was found to be associated with Asterales (Asteraceae) and Asterales (Apiaceae) in addition to Poales (Figure 1, Table 2).

### 3.3. Coevolutionary Testing in Deltocephalinae

**Figure 2:**
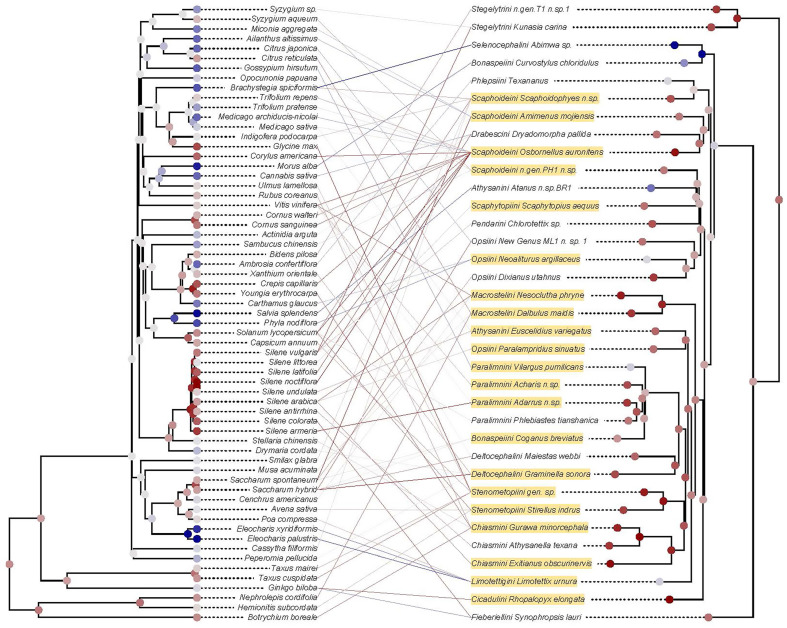
Potential phytoplasma leafhopper vectors (**right**) and their associated host plants (**left**) depicted in a tanglegram produced with the algorithm to maximize incongruence using the Procrustean Approach to Cophylogeny (PACo). The corrected frequencies corresponding to each leafhopper–plant association obtained are mapped using a color scale centered at light grey (zero), ranging from dark red (lowest/incongruent) to dark blue (highest/congruent). The average residual frequency of occurrence of each terminal and fast maximum likelihood estimators of ancestral states of each internal node are also mapped according to the same scale. Specimens confirmed to be infected with phytoplasmas detected in this study are highlighted in yellow. Non-highlighted leafhoppers tested positive for phytoplasma by qPCR but the presence of phytoplasma could not be confirmed from the AHE data obtained.

A total of 82 links between leafhoppers and plants were recorded in our study. The PACo analysis based on patristic distances yielded an m^2^ = 4.54 × 10^6^ with an associated permutational *p* < 0.104, providing evidence for non-significant dependence of the leafhopper phylogeny on the plant phylogeny. Both algorithms, either maximizing congruence or incongruence, yielded similar results (MI algorithm, Figure 2). The bar plots of squared residuals indicate that at least seven links contribute disproportionately to the lack of congruence (Supplementary Figure S2). In leafhoppers, the tribe Chiasmini, generally reported to specialize on grasses, displayed high incongruence between its phylogenetic position and those of their associated plants (internal nodes and terminals in red). In particular, *Gurawa minorcephala*—associated with *Rubus coreanus* and *Botrychium boreale*—and two species associated with *Silene* (Caryophyllaceae), *Exitianus obscurinervis* and *Athysanella texana*, contributed a higher-than-average misfit. Leafhoppers in the tribe Athysanini (*Euscelidius variegatus* and *Atanus* n.sp.BR1) also contributed to incongruence with their associations with *Silene undulata*, *Medicago sativa,* and *Salvia splendens*. The associations that contributed least to the incongruence involved the tribe Stenometopiini (*Stirellus indrus* and gen. sp.) with their associations with plants in the genera *Solanum* (Solanaceae), *Avena* (Poaceae), *Ulmus* (Ulmaceae), and *Taxus* (Taxaceae).

## 4. Discussion

Most previous knowledge of associations between phytoplasmas, their insect vectors, and plant hosts has been compiled through epidemiological studies of phytoplasmas affecting agriculture. However, recent screening of phloem-feeding insect specimens collected from natural areas worldwide indicates that phytoplasmas are ubiquitous in nature and that the diversity of these bacteria is much greater than indicated by the previously documented *Candidatus* Phytoplasma species and 16S groups and subgroups [23,47,48]. As revealed by our results, the diversity of potential host plants and vectors is also much higher than previously documented. We were able to reconstruct food plant diets for 58 specimens, with potential host plants of 45 specimens classified to the genus level or below. Cophylogenetic analysis of the documented associations shows minimal evidence supporting leafhopper–plant cospeciation. Among the 21 specimens included in the cophylogeny that are phytoplasma-infected (Figure 2), these may represent recently acquired plant associations that pose a risk of new outbreaks. Since the precise identification of the detected phytoplasmas is still ongoing using a multilocus approach, we did not attempt a cophylogenetic analysis for phytoplasma and leafhoppers. However, this information will contribute to understanding the complex coevolution of the tripartite associations and better inform models for evaluating the risk of outbreaks.

DNA sequences from the five targeted plant genes—*rbcL*, *matK*, *ITS1*, *ITS2*, and *trnH-psbA*—were successfully recovered from most of the phloem-feeding insects included in our study. Sequences from these different plant loci with variable strengths were used in tandem to identify plants. *ITS1* and *ITS2* had more discriminatory power and were better able to generate species-level identifications, although the high variability of these loci results in lower confidence that classification errors are correctly placed within the same family compared to *matK* and (marginally) *rbcL*. Classification inconsistencies between loci (Supplementary Table S2) may be due to a lack of data for some loci for some plants or variable classification of the same plant due to differences in discriminatory power and database breadth among loci. At the time of writing, the *rbcL* and *matK* databases are the largest, with 361,969 *rbcL*, 302,421 *matK*, 220,400 *ITS1*, 255,007 *ITS2*, and 49,973 *trnH-psbA* sequences available across the plant kingdom in the NCBI database (searching “[all fields]”). Reflected in our choice of maximally consistent genus-level identifications, we note that wild plants are generally under-represented in sequence databases, limiting the identification of potential wild food plants present in the remote collection sites from which the vast majority of our samples were obtained. Incomplete databases can create inconsistencies in the results as the BLAST algorithm tries to find the best, non-existent match, although we found that our stringent filtering approach identified plant taxa with ranges in the sampled areas. Still, additional sampling will likely yield additional results. For species represented by multiple specimens (e.g., *Vilargus pumilicans*) and for specimens for which DNA was submitted for sequencing more than once, there were corroborated results as well as additional classifications added with each molecular sample. It appears that, for many samples, the diversity of plant DNA present was sufficiently sampled, with sequences corroborating each other to yield less-filtered results than the number of long sequences recovered (Supplementary Figure S1), while for others, diet breadth and diversity are likely greater than suggested here, with identification limited by the number of recovered sequences (Section 3.1.2).

Interestingly, our screening of DNA from phytoplasma-infected leafhoppers revealed that many of them ingested DNA from multiple species of plants representing distantly related plant lineages. This is somewhat surprising because many of the tested leafhoppers belong to groups thought to specialize on particular groups of plants. In particular, several tribes of Deltocephalinae are thought to specialize on grasses and related plants (Poales). Many of the tested specimens belonging to these groups (e.g., Chiasmini, Deltocephalini, Paralimnini, and Stenometopiini) indeed yielded DNA sequences from Poales but most also showed evidence of having fed on unrelated plants. This suggests that leafhopper diets are commonly broader than previously suggested, as indicated by statistical tests that revealed little overall congruence between leafhopper and plant (species-level) phylogenies. Chiasmini, one of the presumed grass-specialist lineages, disproportionately contributed to such incongruence alongside Athysanini. Interestingly, one of the included species in the polyphyletic Athysanini, *Euscelidius varigatus*, is in a clade thought to have reversed from grass specialization [7]. While the species-level resolution used to build the phylogeny is limited, it is notable that the majority of the links disproportionately contributing to the lack of congruence were cultivated plants or plants found in insects collected outside their native range, such as *Rubus coreanus* identified in mainland China-collected *Gurawa minorcephala*, and *Silene undulata*, native to southern Africa, identified in Swizterland-collected *Euscelidius variegatus*. While potentially posing an increased risk for phytoplasma spread, this generalized feeding on distantly related plant lineages suggests that other processes, such as host switching [13], may be involved in the observed associations. Over time, the adoption of new hosts may promote or contribute to speciation in some insect groups [49]. Importantly, we have not yet attempted to compare multiple infected and uninfected specimens of the same leafhopper species from the same sample to determine whether phytoplasma infection may be associated with greater diet breadth, but some previous research indicates that phytoplasmas may manipulate their potential vectors in ways that may result in an increase in diet breadth [13,50]. Plants exhibiting symptoms of phytoplasma infection have also been shown to be more attractive to phloem-feeding insects than non-infected plants [51], which may result in feeding by insects that have not yet become infected with phytoplasmas.

Our discovery that some leafhoppers (in the tribes of Chiasmini, Stegelytrini, Deltochephalini, Opsiini, Scaphoideini, Macrostelini, Cicadulini, and Selenocephalini) apparently ingested the DNA of nonvascular plants, including mosses (Pottiales and Hypnales) and algae (Klebsormidiales), warrants further explanation. Some groups of leafhoppers have been observed sucking moisture from wet soil, which may enable them to supplement their diets with minerals difficult to obtain from plant sap. This could provide an explanation for the presence of DNA from algae and moss in some samples.

We identified many tripartite associations—with numerous phytoplasma groups and vectors associated with previously undocumented host plants—that may improve our understanding of the risk of outbreaks. The majority of potential vectors studied (38/58) fed on more than one order; 25 specimens had results in three orders. This wide diet breadth may pose a particular risk for phytoplasma transmission, as such vectors are capable of carrying phytoplasma to additional hosts. To illustrate, in Northwestern Province (Zambia), a *Scaphoidophyes* sp. carrying 16SrXIV-E group phytoplasma presents a particular risk to nearby farms as it was inferred to have fed on plants belonging to nine different orders, including Solanales (*Solananum*). In Madagascar, a species in a new genus (n.gen.) was associated with plants in the genera *Capsicum* (nightshades and cultivated peppers) and *Glycine* (which includes soybeans), potentially putting agriculture at risk if the species proves to be a competent vector. A wide diet breadth was also unexpectedly observed in the ground-dwelling *Conlopa bredoni* (Ulopinae) with unclear dispersal abilities [52]. While phytoplasma infection was not confirmed by AHE data, as the subfamily is not typically associated with phytoplasmas, these results highlight how all groups of phloem-feeding insects can be considered potential vectors even if they do not typically occur in agroecosystems. Among two leafhoppers of the tribe Bonaspeiini, only plants in the family Moraceae, including *Morus* (mulberries), were identified in *Curvostylus chloridulus*, which may pose a risk to regional agriculture (Figure 1A, Table 2).

In North America, the feeding of grass-specialist *Limotettix urnura* on multiple taxa in Poales in Ontario (Canada) presents a particular danger to cereal crops in the region. In the same genus as *Graminella nigrigrons*, a known competent vector of 16SrI phytoplasma [53], *Graminella sonora* carrying 16SrI notably fed on five different orders (Figure 1B, Table 2).

In North and East Asia, numerous species had wide diet breadths and fed on agriculturally relevant genera (Figure 1C, Table 2). *Acharis ussuriensis* (mainland China) fed on plants in a notable nine different orders. Carrying 16Sr XI/XIV-related strain phytoplasma [47,48], it is probable that, as a generalist, the species may also carry other phytoplasma groups; evidence of feeding on *Allium* (including cultivated crops such as onions and garlic) and *Citrus* may indicate particular risks. Likewise, another species collected in mainland China, *Gurawa minorcephala*, was identified as carrying a 16SrXI-related phytoplasma strain and DNA from plants belonging to eight different orders, including *Rubus*, a genus that includes cultivated berries and may pose agricultural risks. Carrying 16SrI-B phytoplasma, the association of *Amemenus mojiensis* with five plant orders, where, alongside Poales (including *Saccharum*), it ingested potentially invasive Myrtales (*Miconia*), reflects a wide and potentially expanding diet breadth. Lastly, Solanales—particularly the genera *Solanum* and *Avena* (Poaceae)—were identified from *Stirellus indrus* (Taiwan). Although the phytoplasma carried by this species could not be identified from the AHE data obtained and may be another bacterium, the insect was collected at the Agricultural Research Institute in Taichung Wufeng and presents the possibility for spillover into these cultivated crops (Figure 1C, Table 2).

In Europe and the Middle East (Figure 1D, Table 2), we inferred a wide diet breadth in the invasive species, *Osbornellus auronitens*, carrying 16SrV-C/D phytoplasma, commonly known as Flavescence dorée phytoplasma (FDp), which has previously been recorded in Switzerland and is associated with important economic losses to grapevines [53]. Interestingly, the collection of *Osbornellus auronitens* in southern Switzerland was conducted during fieldwork carried out by the last author to verify the contribution of natural areas (mixed deciduous woodlands) surrounding commercial vineyards, where outbreaks of FDp have been recorded since 2004 [54]. Previous studies conducted at the same site have reported other alien leafhoppers associated with woody areas harboring FDp-related strains [55,56]. Our present study confirms that *O. auronitens* may represent an additional risk as a potential vector of FDp to nearby vineyards. The risk posed by this species is high because the specimens analyzed in this study also harbored plant DNA from *Vitis* (Figure 1D, Table 2).

In Southeast Asia and Oceania (Figure 1E, Table 2), we found one new genus of Scaphoideini (n.gen.PH2 n.sp.1) at a forested site in the Philippines feeding on native *Opocunonia* trees (Oxidales Cunoniaceae) and Poaceae. As Scaphoideini includes known competent vectors of multiple phytoplasma groups and the tested specimen carries a 16SrXI-related phytoplasma strain that causes outbreaks throughout Asia [57], the inferred host plants may be important to investigate as potential reservoirs. While phytoplasma has previously not been documented in *Maiestas webbi* and the AHE sequence data obtained here were unable to be classified as phytoplasma, the genus includes a known competent vector of 16SrIX phytoplasma, among other groups [58]. The inferred association of *M. webbi* with food plants belonging to Poaceae (*Saccharum*) and Vitaceae (*Vitis*) represents a potential spillover risk to agriculture (Figure 1E, Table 2).

Finally, in South America (Figure 1F, Table 2), the widespread leafhopper *Dalbulus maidis* is a notorious vector of maize bushy stunt phytoplasma (16SrI-B), and it is thought to specialize on corn and its relatives [59]. Interestingly, our specimen, infected with group I phytoplasma and collected in a forested area of the Amazonas (Brazil), did not yield plant DNA identified as Poales, but rather *Citrus* (Rutaceae), *Musa* (Musaceae, bananas, and plantains), and a species in the genus *Nephrolepis* (Nephrolepidaceae). It is possible these plants are reservoir hosts of 16SrI-B phytoplasmas, acting as alternate food plants in areas where the preferred host plant is not available, and/or a concern for spillover. We also identified plants that another supposed grass-specialist leafhopper, *Bolarga nigriloba*, may use as hosts in addition to grasses and detected *Saccharum* DNA in a new species of *Rotundicerus* (French Guiana) infected with 16SrII phytoplasma, presenting a risk to sugarcane crops. 

While more research is needed to deduce which plants are reservoir hosts and the extent to which phytoplasma infection influences the diet breadth of potential vectors, these tripartite results help fill significant knowledge gaps in the ecology of the phytoplasma–vector–plant system. Transmission trials and increased sampling across the diversity of Cicadellidae, particularly in natural areas, will help elucidate additional phytoplasma plant hosts and insect vectors. Future studies determining the extent to which phytoplasmas may induce changes in the diet breadth of their vectors are necessary to further disentangle the system’s evolutionary history and prevent phytoplasma disease outbreaks.

## 5. Conclusions

Next-generation sequencing approaches provide efficient tools for documenting associations between phytoplasmas, their potential insect vectors, and host plants. By screening DNA extracted non-destructively from the bodies of individual phloem-feeding insects, we can not only identify potential phytoplasma vectors but also infer their food plant preferences while retaining the insect’s exoskeleton as a voucher specimen useful for further documenting the identity of the potential phytoplasma vector. This approach is particularly relevant for disentangling complex epidemiological cycles involving highly mobile, polyphagous insect vectors inhabiting adjacent agroecosystems and natural areas. Wild plant DNA is still poorly represented in public sequence databases, and, at present, this may limit our ability to identify the food plants of potential phytoplasma vectors with high precision. Nevertheless, our analysis of DNA extracted from whole bodies of individual leafhoppers and cophylogenetic analysis suggest that potential vectors (leafhoppers) infected with phytoplasmas fed on a greater diversity of plant species than expected based on their membership in groups thought to have more restricted diets (e.g., putative grass specialists) and the phylogenetic relatedness of their inferred food plants. Our results suggest that leafhoppers may feed on multiple phylogenetically unrelated plants and, coincidentally, acquire phytoplasma pathogens within a short period during their lifespan, which may enhance the potential for transmission of phytoplasmas among potential plant hosts and spillover from natural vegetation into agroecosystems. Our results highlight the need for more research on phytoplasma-mediated plant host switches to better explain the real risk of unexpected but still predictable outbreaks.

## Figures and Tables

**Table 1:** Summary of recovered plant sequences for five barcoding genes across 75 assessed samples.

	ITS1	ITS2	matK	rbcL	trnH-psbA
Total Recovered Sequences (=939)	280	166	141	325	27
Average Sequence Length	143.6	168.5	254.8	245.8	164.1
Complete Sequences (%)	10.7	1.2	0.0	0.0	7.4
Samples with Sequences (%)	70.7	66.7	57.3	84.0	24.0
Average Sequences Per Sample	5.3	3.3	3.3	5.2	1.5
Median Sequences Per Sample	3.0	2.0	2.0	3.0	1.0
Classification Supported to Genus Level (%) ^1^	58.2	60.2	45.4	29.2	48.1
Classification Supported to Family Level (%) ^1^	85.7	65.1	100.0	88.0	85.2
Classification Supported to Order Level (%) ^1^	93.6	86.7	100.0	95.7	88.9
Unique Supported Genera ^1^	39	42	29	37	10
Unique Supported Families ^1^	29	29	31	45	10
Unique Supported Orders ^1^	21	24	24	33	10
Total Unique Supported Results ^1^	52	51	49	81	13

^1^ Supported classification indicates >95% of BLAST results were in agreement at that classification level, considering only results with >98% identity and within 1% of the highest percent identity result for that sequence.

**Table 2:** Summarized tripartite results for each specimen (n = 58). Identified plants include classifications to the order, family, and/or genus. The last column includes the phytoplasma classification at seven loci.

Geographic Area|Country or Region ^1^	Tribe	Species	Identified Plants (Order;Family;Genus) ^2^	Phytoplasma (16Sr|Tuf|SecA|SecY|rpIV-rpsC|groEl) ^3^
A | Eswatini	Ulopini	* Conlopa bredoni *	Commelinales;Commelinaceae;None Commelinales;Commelinaceae;Commelina Fabales;Fabaceae;None Poales;Poaceae;Aristida Poales;Poaceae;Oropetium Fabales;Fabaceae;Vigna Lamiales;Lamiaceae;None	-|oBa|oBa|-|-|-
A | Madagascar	Scaphoideini	n.gen.MG5 n.sp.1	Hypnales;None;None Hypnales;Neckeraceae;None	16SrVI|-|-|-|-|-
A | Republic of Congo	Scaphoideini	n.gen.ZA5 n.sp.1	Vitales;Vitaceae;None	-|oBa|oBa|-|-|-
A | Madagascar	Stenometopiini	n.gen. sp.	Cupressales;Taxaceae;Taxus Rosales;Ulmaceae;None Rosales;Ulmaceae;Ulmus Solanales;Solanaceae;None Solanales;Solanaceae;Capsicum Fabales;Fabaceae;Glycine	-|oBa|IV|-|-| algae
A | South Africa	Bonaspeiini	* Curvostylus chloridulus *	Rosales;Moraceae;None Rosales;Moraceae;Morus	NA
A | South Africa	Bonaspeiini	* Coganus breviatus *	Poales;Poaceae;None Poales;Poaceae;Saccharum Zingiberales;None;None Apiales;Apiaceae;None	16SrXI|XI|XI|V/XI|XIV/XI
A | South Africa	Paralimnini	* Vilargus pumilicans *	Poales;Poaceae;None	-|XI|VIII|na|-|XI
A | South Africa	Paralimnini	* Vilargus pumilicans *	Asterales;Asteraceae;None Poales;Poaceae;None Malvales;Malvaceae;Gossypium	16SrX|oBa|VIII|-|V|XI
A | Zambia	Stenometopiini	*Stirellus* sp.	Poales;Poaceae;Cenchrus	NA
A | Zambia	Scaphoideini	*Scaphoidophyes* n.sp.	Fabales;Fabaceae;Brachystegia Asterales;Asteraceae;None Fabales;Fabaceae;None Gentianales;Apocynaceae;None Laurales;Lauraceae;Cassytha Sapindales;Rutaceae;None Solanales;Solanaceae;Solanum Fabales;Fabaceae;Indigofera Liliales;Smilacaceae;Smilax Zingiberales;None;None Lamiales;Verbenaceae;None	16SrXIV-E |-|-|-|-|-
A | Zambia	Selenocephalini	*Abimwa* sp.	Klebsormidiales;Klebsormidiaceae;Klebsormidium Hypnales;None;None Fabales;Fabaceae;Brachystegia	-|oBa|-|-|-|-
EM | France	Fieberiellini	* Synophropsis lauri *	Caryophyllales;Caryophyllaceae;Silene Ginkgoales;Ginkgoaceae;Ginkgo Poales;Poaceae;None	NA
EM | Israel	Opsiini	* Neoaliturus argillaceus *	Asterales;Asteraceae;Carthamus Caryophyllales;Polygonaceae;None Lamiales;Verbenaceae;Phyla	16SrIX-J|oBa|V|IX|IX|XIV
EM | Switzerland	Scaphoideini	* Osbornellus auronitens *	Asterales;Asteraceae;None Asterales;Asteraceae;Crepis Caryophyllales;Caryophyllaceae;None Caryophyllales;Caryophyllaceae;Silene Cornales;Cornaceae;Cornus Fabales;Fabaceae;None Fabales;Fabaceae;Medicago Fabales;Fabaceae;Trifolium Fagales;Betulaceae;None Fagales;Betulaceae;Corylus Rosales;Rhamnaceae;None Sapindales;Rutaceae;None Solanales;None;None Solanales;Solanaceae;Solanum Vitales;Vitaceae;None Vitales;Vitaceae;Vitis Commelinales;Commelinaceae;None Ericales;None;None	16SrV-C|V-C/D|16SrV-C/D|16SrV-C/D|V|V-C/D
EM | Switzerland	Cicadulini	* Rhopalopyx elongata *	Hypnales;None;None Ginkgoales;Ginkgoaceae;Ginkgo	16SrVI-L|V-C/D|V|VI|VI|V
EM | Switzerland	Athysanini	* Euscelidius variegatus *	Caryophyllales;Caryophyllaceae;None Fabales;Fabaceae;None Fabales;Fabaceae;Medicago Caryophyllales;Caryophyllaceae;Silene	16SrI-F|I|I|I|I|I
NA | Canada	Limotettigini	* Limotettix urnura *	Poales;Cyperaceae;None Poales;Cyperaceae;Eleocharis Poales;Poaceae;Poa	16SrXI-G |V|XI|-|-|XI
NA | Mexico	Phlepsiini	* Texananus ovatus *	Asterales;Asteraceae;None	NA
NA | Mexico	Phlepsiini	*Texananus* [nymph]	Asterales;Asteraceae;None Asterales;Asteraceae;Ambrosia	NA
NA | Mexico	Scaphytopiini	* Scaphytopius aequus *	Solanales;Solanaceae;None Caryophyllales;Caryophyllaceae;Silene	-|I|I|I|I|I
NA | USA	Deltocephalini	* Graminella sonora *	Asterales;Asteraceae;None Asterales;Asteraceae;Xanthium Caryophyllales;Caryophyllaceae;None Caryophyllales;Caryophyllaceae;Drymaria Piperales;Piperaceae;Peperomia Poales;Poaceae;None Poales;Poaceae;Saccharum Rosales;Moraceae;None	-|I|I|I|I|I
NA | USA	Chiasmini	* Athysanella texana *	Caryophyllales;Caryophyllaceae;Silene	NA
NA | USA	Opsiini	* Dixianus utahnus *	Fabales;Fabaceae;None Poales;Poaceae;None Poales;Poaceae;Saccharum	-|-|algae|-|-|-
NEA | Mainland China	Stenometopiini	* Stirellus productus *	Poales;Poaceae;None Fagales;Fagaceae;None	-|XI|XI|XI|XI|XI
NEA | Mainland China	Chiasmini	* Gurawa minorcephala *	Asterales;Asteraceae;Youngia Caryophyllales;Caryophyllaceae;None Caryophyllales;Polygonaceae;None Hypnales;None;None Poales;Poaceae;None Pottiales;Pottiaceae;None Rosales;Rosaceae;Rubus Caryophyllales;Caryophyllaceae;Stellaria Ophioglossales;Ophioglossaceae;Botrychium	16SrXI|XI|XI|XI|XI|XI
NEA | Mainland China	Hecalini	* Fangamanus tripunctatus *	Malvales;Malvaceae;None Poales;Poaceae;None	-|oBa|oBa|-|-|-
NEA | Mainland China	Paralimnini	*Acharis* n.sp.	Poales;Poaceae;None Vitales;Vitaceae;None Vitales;Vitaceae;Vitis	-|oBa|XI|-|-|-
NEA | Mainland China	Paralimnini	* Acharis ussuriensis *	Rosales;Cannabaceae;Celtis Asparagales;Amaryllidaceae;Allium Asparagales;Amaryllidaceae;None Pinales;Pinaceae;Pinus Sapindales;Rutaceae;Citrus Apiales;Apiaceae;None Fagales;Fagaceae;None Asparagales;None;None Asterales;Asteraceae;None Asterales;Asteraceae;Bidens Laurales;Lauraceae;None Poales;Poaceae;None Poales;Poaceae;Brachypodium Sapindales;Rutaceae;None Fabales;Fabaceae;Phaseolus	16SrXIV|oBa|XI/I|V/XI|V|XIV/I
NEA | Mainland China	Scaphoideini	* Amimenus mojiensis *	Dipsacales;None;None Poales;Poaceae;None Poales;Poaceae;Saccharum Sapindales;Simaroubaceae;Ailanthus Myrtales;Melastomataceae;Miconia	16SrI-B|I|I|I|I|I
NEA | Kyrgyzstan	Paralimnini	* Phlebiastes tianshanica *	Solanales;Solanaceae;Solanum Apiales;Apiaceae;None	NA
NEA | Mongolia	Paralimnini	*Adarrus* n.sp.	Asterales;None;None Caryophyllales;Caryophyllaceae;None Vitales;Vitaceae;None Vitales;Vitaceae;Vitis Caryophyllales;Caryophyllaceae;Silene Sapindales;Rutaceae;None	-|oBa|-|-|-|IX
NEA | Taiwan	Stenometopiini	* Stirellus indrus *	Poales;Poaceae;Avena Polypodiales;Pteridaceae;None Solanales;Solanaceae;Solanum Polypodiales;Pteridaceae;Hemionitis	-|oBa|-|-|-|-
NEA | Taiwan	Drabescini	* Dryadomorpha pallida *	Ericales;Actinidiaceae;None Ericales;Actinidiaceae;Actinidia Rosales;Cannabaceae;Cannabis Caryophyllales;Caryophyllaceae;Silene	NA
NEA | Taiwan	Opsiini	* Alishania formosana *	Fagales;Fagaceae;None Fagales;None;None	new group|XXI|IV|IV|IV|IV
SA | Argentina	Deltocephalini	* Bolarga nigriloba *	Apiales;Apiaceae;None Asterales;Asteraceae;None Poales;Poaceae;None	16SrXI-B|-|-|-|-|-
SA | Brazil	Athysanini	*Atanus* n.sp.BR1	Lamiales;Lamiaceae;Salvia	NA
SA | Brazil	Pendarini	*Chlorotettix* sp.	Caryophyllales;Caryophyllaceae;Silene	-|oBa|-|-|-|-
SA | Brazil	Deltocephalini	*Cortona* n.sp.	Pottiales;Pottiaceae;None	NA
SA | Brazil	Macrostelini	* Dalbulus maidis *	Sapindales;Rutaceae;None Sapindales;Rutaceae;Citrus Zingiberales;Musaceae;Musa Polypodiales;Nephrolepidaceae;Nephrolepis	-|I|I|I|I|I
SA | French Guiana	Chiasmodolini	*Rotundicerus* n.sp.	Caryophyllales;Caryophyllaceae;Silene Poales;Poaceae;None Poales;Poaceae;Saccharum Cupressales;Cupressaceae;None	16SrIII|-|-|-|-|-
SA | Peru	Chiasmini	* Exitianus obscurinervis *	Caryophyllales;Caryophyllaceae;Silene	16SrIII|oBa|-|-|-|-
SA | Peru	Bahitini	* Taperinha adspera *	Dipsacales;Adoxaceae;Sambucus Oxalidales;Cunoniaceae;None Oxalidales;Cunoniaceae;Opocunonia Myrtales;Myrtaceae;Syzygium	NA
SEAO | Australia	Deltocephalini	* Maiestas webbi *	Poales;Poaceae;None Poales;Poaceae;Saccharum Vitales;Vitaceae;Vitis	NA
SEAO | Australia	Macrostelini	* Nesoclutha phryne *	Poales;Poaceae;None Rosales;Ulmaceae;None Vitales;Vitaceae;None Vitales;Vitaceae;Vitis Caryophyllales;Caryophyllaceae;Silene Poales;Poaceae;Tripogonella Poales;Poaceae;Cenchrus	16SrXIV|oBa|XI|na|-|XI
SEAO | Australia	Macrostelini	* Nesoclutha phryne *	Vitales;None;None Vitales;Vitaceae;None Vitales;Vitaceae;Vitis	NA
SEAO | Australia	Scaphoideini	*Diemoides* n.sp.1	Solanales;Solanaceae;None	16SrXIV-D|-|oBa|-|-|-
SEAO | Malaysia	Stegelytrini	* Kunasia carina *	Caryophyllales;Caryophyllaceae;Silene Myrtales;None;None Myrtales;Myrtaceae;None Myrtales;Myrtaceae;Syzygium Pottiales;Pottiaceae;None	NA
SEAO | Malaysia	Opsiini	New Genus ML1 n.sp.1	Malvales;Dipterocarpaceae;None Poales;Poaceae;None Poales;Poaceae;Saccharum Sapindales;Rutaceae;None Sapindales;Rutaceae;Citrus	-|oBa|-|-|-|-
SEAO | Philippines	Scaphoideini	n.gen.PH2 n.sp.1	Oxalidales;Cunoniaceae;Opocunonia Poales;Poaceae;None	16SrXI|-|-|-|-
SEAO | Philippines	Drabescini	n.gen.PH3 n.sp.1	Caryophyllales;Caryophyllaceae;Silene	NA
SEAO | Philippines	Megipocerini	*Chunra* n.sp.PH1	Rosales;Moraceae;None Rosales;Moraceae;Morus Rosales;Rosaceae;Prunus Fabales;Fabaceae;Trifolium	16SrXIV|-|-|-|-|-
SEAO | Thailand	Paralimnini	* Multiproductus *	Poales;Poaceae;None Poales;Poaceae;Saccharum Vitales;Vitaceae;None Vitales;Vitaceae;Vitis	16SrXIV-E|oBa|-|-|I|-
SEAO | Thailand	Stegelytrini	n.gen.T1 n.sp.1	Hypnales;None;None Hypnales;Neckeraceae;None Poales;Poaceae;None Hypnales;Neckeraceae;Thamnobryum Asterales;Asteraceae;Bidens	NA
SEAO | Thailand	Scaphoideini	*Scaphomonus* n.sp.T1	Hypnales;None;None Hypnales;Neckeraceae;None	16SrV|-|-|V|-|-
SEAO | Thailand	Opsiini	* Paralampridius sinuatus *	Caryophyllales;Caryophyllaceae;None Caryophyllales;Caryophyllaceae;Silene Poales;Poaceae;None Pottiales;Pottiaceae;None	-|-|-|-|I|-
SEAO | Thailand	Opsiini	New Genus T3 n.sp.1	Caryophyllales;Caryophyllaceae;None	NA
SEAO| Fiji	Opsiini	* Navaia filicola *	Poales;Poaceae;None	-|I|-|-|-|-
SEAO| Fiji	Macrostelini	*Balclutha* n.sp.FI1	Hypnales;None;None Hypnales;Neckeraceae;None Hypnales;Neckeraceae;Thamnobryum Poales;Poaceae;None	-|oBa|-|-|-|-

^1^ Leafhoppers are grouped into six geographic areas (A = Africa, EM = Europe and the Middle East, NA = North America, NEA = North and East Asia, SA = South America, and SEAO = Southeast Asia and Oceania). ^2^ “None” indicates that the sequence(s) could not be classified with high confidence to a single taxon at that level. ^3^ oBa = BLASTing to other Bacteria, - = classification unclear or not available on the corresponding locus, NA = no data.

## Data Availability

The R scripts used to generate the results for this study are publicly available at https://github.com/amgabrys/PhytoplasmaTripartiteAssociations_2024 (accessed on 11 January 2025).

## Supplementary Materials

The following are available online at https://www.mdpi.com/article/10.3390/pathogens14010074/s1, Figure S1: Role of recovered sequence count in determining diet diversity. Samples sorted by number of total filtered results; Figure S2: Jack-knifed squared residuals (bars) and upper 95% confidence intervals (error bars) associated with each plant–leafhopper link. PACo was applied to patristic distances. The dashed line indicates the median squared residual value. Table S1: Filtered and summarized BLAST results for sequences obtained using the Anchored Hybrid Enrichment approach for each leafhopper specimen that tested positive for phytoplasma presence; Table S2: Summarized BLAST results for each recovered plant sequence obtained using the Anchored Hybrid Enrichment approach; Table S3: Leafhopper–plant associations tested for cophylogentic analysis using a global fit approach. Type indicates how the insect phylogeny (Cao et al., 2022 [7]) was subset for that sample, where FromILL_137401_ID indicates that molecular data from that exact specimen was included in the phylogeny, MatchedInsectTreewGenusSpecies indicates that the specimen is represented by a leafhopper of the same species, and MatchedInsectTreewTribeGenus indicates that the specimen is represented by a leafhopper of the same genus.
