# Supplementary figures and images for: Inferring Tripartite Associations of Vector-Borne Plant Pathogens Using a Next-Generation Sequencing Approach
Source: Pathogens. 2025 Jan 14;14(1):74. doi: 10.3390/pathogens14010074 (PMC11768818; doi:10.3390/pathogens14010074)

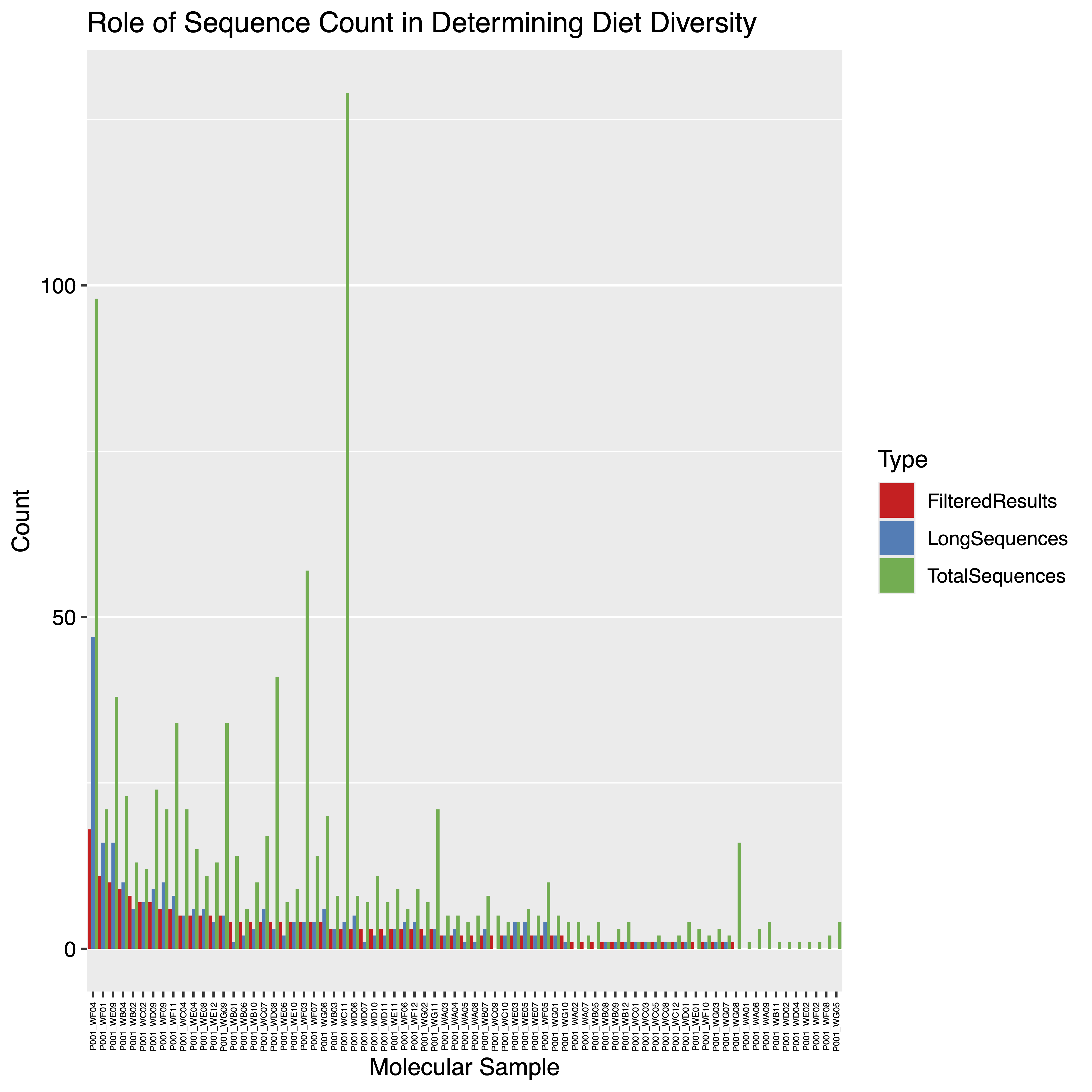

Supplement: Supplementary file 1 [file pathogens-14-00074-s001.zip › Supplementary Figure S1.png]

Squared residuals

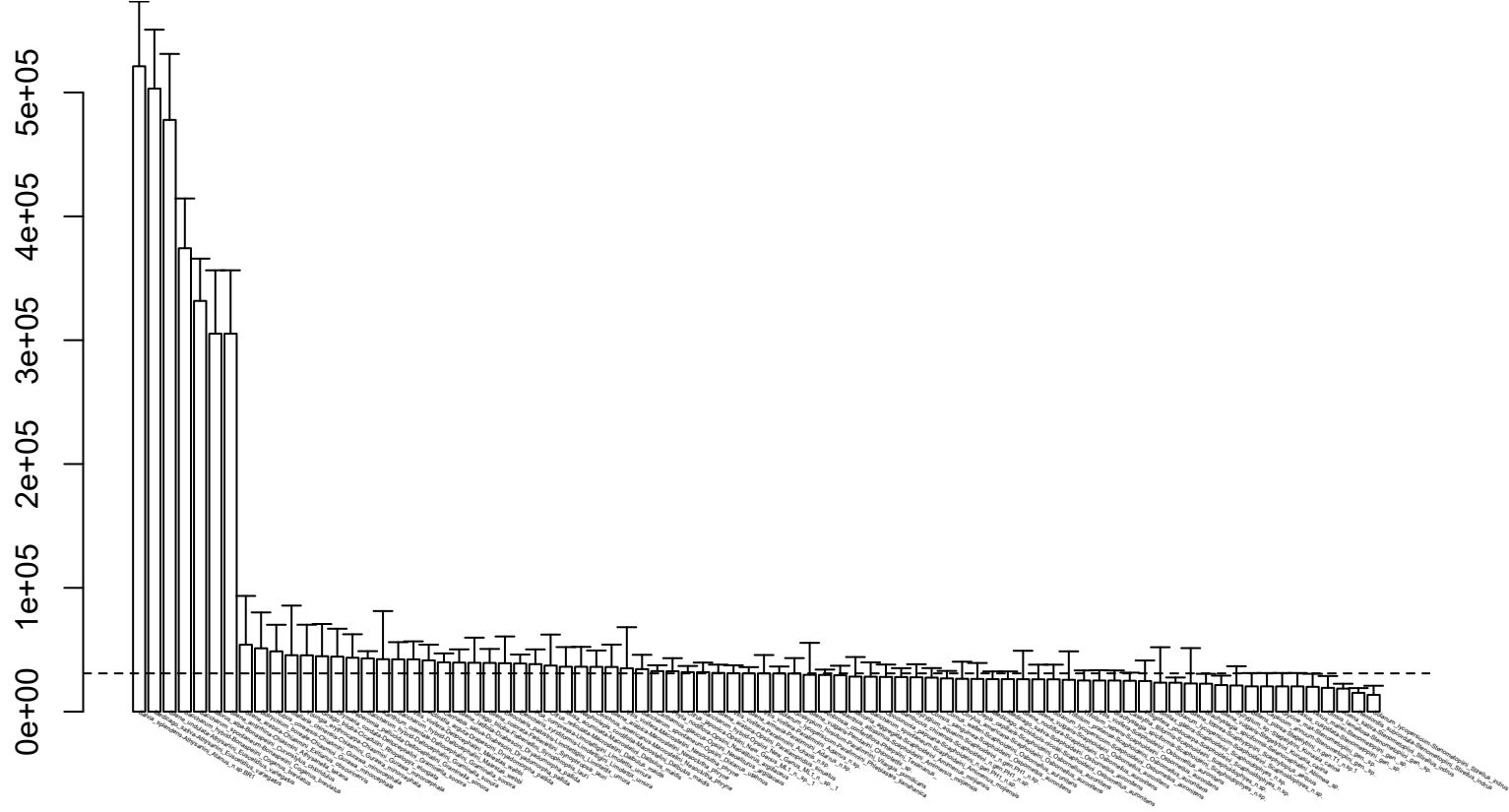

Supplement: Supplementary file 1 [file pathogens-14-00074-s001.zip › Supplementary Figure S2.pdf]
